# Prospective multi-center registry to evaluate efficacy and safety of the newly developed diamond-like carbon-coated cobalt–chromium coronary stent system

**DOI:** 10.1007/s12928-016-0407-z

**Published:** 2016-07-22

**Authors:** Kenji Ando, Katsuhisa Ishii, Eiji Tada, Kazuaki Kataoka, Atsushi Hirohata, Kenji Goto, Katsuyuki Kobayashi, Hiroshi Tsutsui, Makoto Nakahama, Hitoshi Nakashima, Shinichiroh Uchikawa, Junji Kanda, Satoshi Yasuda, Junji Yajima, Hiroshi Kitabayashi, Shumpei Sakurai, Keita Nakanishi, Naoto Inoue, Hirofumi Noike, Terumitsu Hasebe, Tetsuya Sato, Masao Yamasaki, Takeshi Kimura

**Affiliations:** 1 Department of Cardiology, Kokura Memorial Hospital, 3-2-1 Asano, Kokurakita, Kitakyushu, Fukuoka 802-8555 Japan; 2 Department of Cardiology, Kansai Electric Power Hospital, Osaka, Japan; 3 Department of Cardiology, Nabari City Hospital, Nabari, Japan; 4 Department of Cardiology, The Sakakibara Heart Institute of Okayama, Okayama, Japan; 5 Department of Cardiovascular Medicine, Fukuyama Cardiovascular Hospital, Fukuyama, Japan; 6 Department of Cardiovascular Medicine, Ageo Central General Hospital, Ageo, Japan; 7 Department of Cardiovascular Medicine, Suwa Red Cross Hospital, Suwa, Japan; 8 Department of Cardiology, Fukuyama City Hospital, Fukuyama, Japan; 9 Department of Cardiovascular Medicine, National Hospital Organization Kagoshima Medical Center, Kagoshima, Japan; 10 Department of Cardiology, Azumino Red Cross Hospital, Azumino, Japan; 11 Department of Cardiovascular Medicine, Asahi General Hospital, Asahi, Japan; 12 Department of Cardiovascular Medicine, National Cerebral and Cardiovascular Center, Osaka, Japan; 13 The Cardiovascular Institute, Tokyo, Japan; 14 Department of Cardiovascular Medicine, Ina Central Hospital, Ina, Japan; 15 Department of Cardiology, Marunouchi Hospital, Tokyo, Japan; 16 Department of Internal Medicine, Ibaraki Seinan Medical Center Hospital, Sashima, Japan; 17 Department of Cardiology, Sendai Kousei Hospital, Sendai, Japan; 18 Department of Cardiovascular Medicine, Toho University Medical Center Sakura Hospital, Sakura, Japan; 19 Department of Radiology, Tokai University Hachioji Hospital, Hachioji, Japan; 20 Department of Cardiovascular Medicine, Japanese Red Cross Okayama Hospital, Okayama, Japan; 21 Department of Cardiology, NTT Medical Center Tokyo, Tokyo, Japan; 22 Department of Cardiovascular Medicine, Kyoto University Graduate School of Medicine, Kyoto, Japan

**Keywords:** Coronary stent, Restenosis, Thrombosis, Diamond-like carbon (DLC)

## Abstract

The purpose of this multi-center, non-randomized, and open-label clinical trial was to determine the non-inferiority of diamond-like carbon (DLC)-coated cobalt–chromium coronary stent, the MOMO DLC coronary stent, relative to commercially available bare-metal stents (MULTI-LINK VISION^®^). Nineteen centers in Japan participated. The study cohort consisted of 99 patients from 19 Japanese centers with single or double native coronary vessel disease with de novo and restenosis lesions who met the study eligibility criteria. This cohort formed the safety analysis set. The efficacy analysis set consisted of 98 patients (one case was excluded for violating the eligibility criteria). The primary endpoint was target vessel failure (TVF) rate at 9 months after stent placement. Of the 98 efficacy analysis set patients, TVF occurred in 11 patients (11.2 %, 95 % confidence interval 5.7–19.2 %) at 9 months after the index stent implantation. The upper 95 % confidence interval for TVF of the study stent was lower than that previously reported for the commercially available MULTI-LINK VISION^®^ (19.6 %), demonstrating non-inferiority of the study stent to MULTI-LINK VISION^®^. All the TVF cases were related to target vascular revascularization. None of the cases developed in-stent thrombosis or myocardial infarction. The average in-stent late loss and binary restenosis rate at the 6-month follow-up angiography were 0.69 mm and 10.5 %, respectively, which are lower than the reported values for commercially available bare-metal stents. In conclusion, the current pivotal clinical study evaluating the new MOMO DLC-coated coronary stent suggested its low rates of TVF and angiographic binary restenosis, and small in-stent late loss, although the data were considered preliminary considering the small sample size and single arm study design.

## Introduction

Percutaneous coronary intervention (PCI) with a coronary stent for coronary artery disease has remarkably reduced complications, such as acute occlusion of the coronary artery and restenosis. As a result, the metallic coronary stent has been widely used for treating coronary artery disease. However, implanting a metallic stent in the coronary artery induces proliferative and inflammatory reactions because of damage to the vessel wall and/or reactions to foreign materials [1–3]. This results in neointimal hyperplasia during healing process, which in turn causes restenosis of the coronary artery in 14–20 % of patients who undergo coronary bare-metal stent (BMS) implantation. To prevent such restenosis after stent implantation, several drug-eluting stents (DESs) have been developed in recent years. However, restenosis still occurs after DES implantation, and the target vessel failure (TVF) rate ranges 3–8 % [4, 5]. Despite introduction of DES, BMS is still used in 10–20 % of patients undergoing PCI (JROAD: The Japanese Registry of All cardiac and vascular Diseases, http://www.j-circ.or.jp/jittai_chosa/).

The first metallic coronary stent to be approved in Japan was the Palmaz-Schatz stent manufactured by Johnson & Johnson and made from 316-L stainless steel alloy. Stainless steel is poorly visible because of its low radio-opacity. Therefore, it is difficult to confirm the location of this stainless steel coronary stent under the angiography. Furthermore, because the radial force of the stainless steel coronary stent is not sufficiently strong, the strut thickness of the stainless steel coronary stent exceeds 100 μm. This leads to inferior crossability and a higher restenosis rate. To solve these problems, cobalt–chromium stents, such as “Driver” (from Medtronic) and “MULTI-LINK VISION^®^” (from Abbott Vascular), have been developed. These stents have better visibility and a higher radial force than stainless steel coronary stents. However, these cobalt–chromium alloy coronary stents were also reported to be associated with relatively high restenosis rates.

**Fig. 1:**
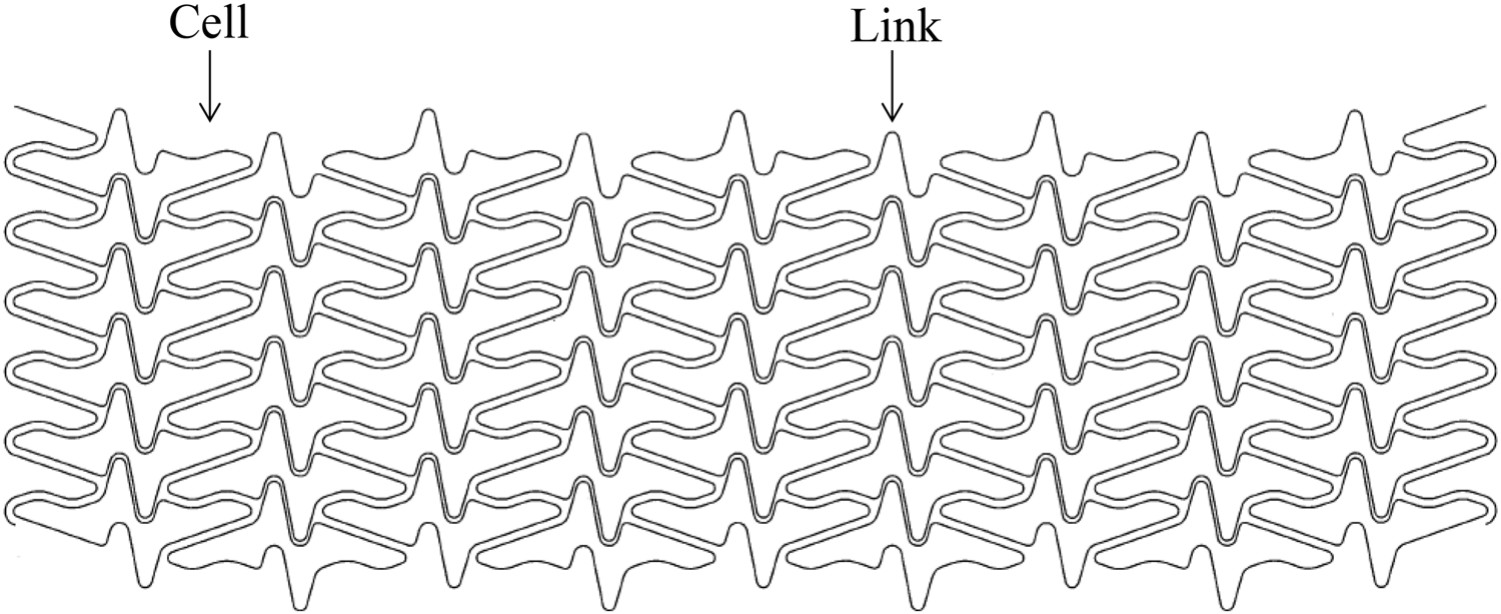
Schematic representation of the MOMO DLC-coated coronary stent design

To overcome the shortcomings of conventional BMS, the MOMO diamond-like carbon (DLC)-coated coronary stent was developed by Japan Stent Technology Co., Ltd. in collaboration with Fukuda Denshi Co., Ltd. in Japan. Coating the cobalt–chromium material of a stent with DLC might reduce thrombosis and corrosion, as expected from the results of the non-clinical tests of the MOMO DLC-coated coronary stent. For this reason, DLC coating is used for devices employed in the fields of cardiology and orthopedics [6, 7]. It has also been reported that when DLC coats a metallic material, it prevents ion elution from the metallic surface. Gutensohn et al. reported that DLC coating actually prevent ion elution from coronary stent [8]. The MOMO DLC-coated coronary stent is also characterized by its thin strut thickness of 70 µm, which is one of the thinnest commercially available coronary stents at this moment. Mongrain and Kastrati et al. reported that a thinner stent strut can reduce the shear stress and restenosis rate [9, 10]. Although stents with such thin strut thicknesses are generally thought to have insufficient radial force, the MOMO DLC-coated coronary stent overcomes this problem through the strength of its cobalt–chromium alloy material and the closed cell design of its linked cells. As a result, the MOMO DLC-coated coronary stent maintains high radial force without sacrificing its conformability and longitudinal strength (Fig. 1).

Therefore, we conducted a prospective multi-center registry to evaluate the safety and efficacy of the MOMO DLC-coated coronary stent relative to the commercially available BMS as the pivotal clinical study to get regulatory approval of this new coronary stent in the Japanese market.

## Methods

### Study design

This clinical study was conducted among 19 centers in Japan from October 2010 to November 2012. Patients were eligible to enter the study if they had single or double vessel coronary artery disease with objective signs and/or symptoms of myocardial ischemia. We enrolled patients with maximum of 2 lesions for PCI, both de novo and restenosis, with reference diameters of 3.0–4.0 mm, and lesion length ≤26 mm. The major exclusion criteria included acute myocardial infarction within 72 h, in-stent restenosis, and previous DES implantation in the target vessel. When a patient has 2 lesions scheduled for PCI, the operator should declare which lesion is the target lesion for the current study evaluating the MOMO DLC-coated coronary stent stents. The non-target lesion should be treated first with use of any coronary stents commercially available, including DES, while the target lesion should be treated with the study stents if PCI for the non-target lesion was completed uneventfully.

The MOMO DLC-coated coronary stents were available in diameters of 3.0, 3.5, and 4.0 mm with each available in lengths of 8, 13, 18, 23, and 28 mm for the current study. Clinical follow-up was to be conducted at 1, 3, 6, and 9 months after the procedure. Angiography was scheduled at the 6-month follow-up in all patients, and quantitative coronary angiography (QCA) was performed by an angiographic core laboratory (Cardiocore Japan).

Dual antiplatelet therapy (DAPT) consisting of aspirin and clopidogrel was mandatory for at least 1 month, and the duration of DAPT beyond 1 month was left to the discretion of the attending physicians.

The study was conducted according to the Declaration of Helsinki. The study protocol was approved by the institutional review board in each participating center, and written informed consent was obtained from all the participants.

### Endpoints

The primary endpoint was the rate of TVF through 9-month follow-up, defined as a composite of cardiac death, myocardial infarction (MI), and clinically indicated target vessel revascularization (TVR). TVR was defined as PCI or coronary artery bypass grafting (CABG) for the target vessel. A TVR was considered clinically indicated if one of the following occurred: (1) a positive history of recurrent angina pectoris, presumably related to the target vessel; (2) objective signs of ischemia at rest (ECG changes) or during exercise test (or equivalent), presumably related to the target vessel; (3) abnormal results of any invasive functional diagnostic test (e.g., fractional flow reserve); (4) a target lesion revascularization with a diameter stenosis greater than 70 % even in the absence of the above-mentioned ischemic signs or symptoms.

The secondary endpoints included: (1) TVF rate at the 6-month follow-up, (2) in-stent late loss index, as determined by QCA, (3) in-stent binary restenosis rate as defined by % diameter stenosis ≥50 %, as determined by angiography, and (4) the acute procedural success rate.

### Statistical analysis

Categorical variables were expressed as number and percentage, and continuous variables as mean value ± standard deviation. The incidence of the endpoint event was calculated as the percentage of patients with the event in those patients who completed 9-month follow-up.

This study was designed to demonstrate that the MOMO DLC-coated coronary stent was non-inferior to the commercially available MULTI-LINK VISION^®^ coronary stent in terms of TVF at 9 months. For the MULTI-LINK VISION^®^, TVF rate at 9 months was reported to be 14.7 % [95 % confidence intervals (CIs) 10.6–19.6 %] [11]. Non-inferiority of the MOMO DLC-coated coronary stent to the MULTI-LINK VISION^®^ could be declared if the upper 95 % CI of TVF in the current study is lower than that of the MULTI-LINK VISION^®^ (19.6 %). With alpha error of 5 %, enrollment of 88 patients would provide 80 % power to demonstrate non-inferiority of the MOMO DLC-coated coronary stent to the MULTI-LINK VISION^®^. Considering 10 % dropout during follow-up, the sample size for the current study consisted of 100 patients.

## Results

### Patient characteristics

**Fig. 2:**
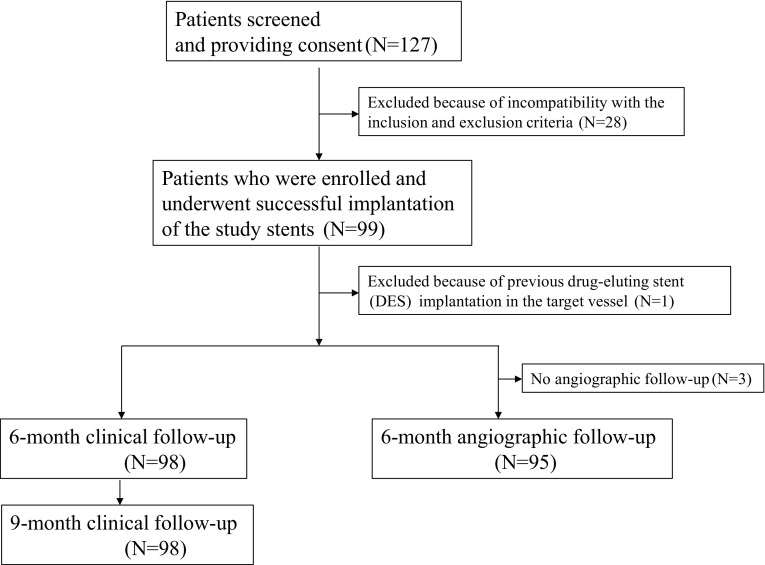
Study flow chart of the trial profile

In total, 127 patients were screened and provided written informed consent to participate in this study, but 28 patients were excluded, because they did not meet the angiographic inclusion criteria. Therefore, 99 patients were enrolled in this study, and the study stents were successfully implanted in all patients (Fig. 2). The clinical and angiographic characteristics of the patients enrolled in this study were unremarkable and generally comparable with most of the pivotal studies of BMS in Japan (Table 1). The efficacy of the study device was evaluated in the per-protocol population (98 patients) after excluding 1 patient in whom DES had already been implanted in the target vessel (exclusion criteria). In these 98 patients, the study stents with the smallest diameter (3.0 mm) and the longest length (28 mm) were used in 38 patients (38.4 %) and in 10 patients (10.1 %), respectively.

**Table 1:** Clinical and angiographic characteristics

Number of patients	99
Male	84 (85 %)
Age (years: mean ± SD)	68.6 ± 9.3
Hypertension	79 (80 %)
Diabetes	36 (36 %)
Hypercholesterolemia	80 (81 %)
History of smoking	71 (72 %)
Family history of CAD	20 (20 %)
Prior myocardial infarction	28 (28 %)
Prior PCI	35 (35 %)
Prior CABG	3 (3 %)
Stable CAD	85 (86 %)
Unstable angina	14 (14 %)
Target vessel–LAD	35 (35 %)
Target vessel–LCX	22 (22 %)
Target vessel–RCA	42 (42 %)

*SD* standard deviation, *CAD* coronary artery disease, *PCI* percutaneous coronary intervention, *CABG* coronary artery bypass grafting, *LAD* left anterior descending coronary artery, *LCX* left circumflex coronary artery, *RCA* right coronary artery

**Fig. 3:**
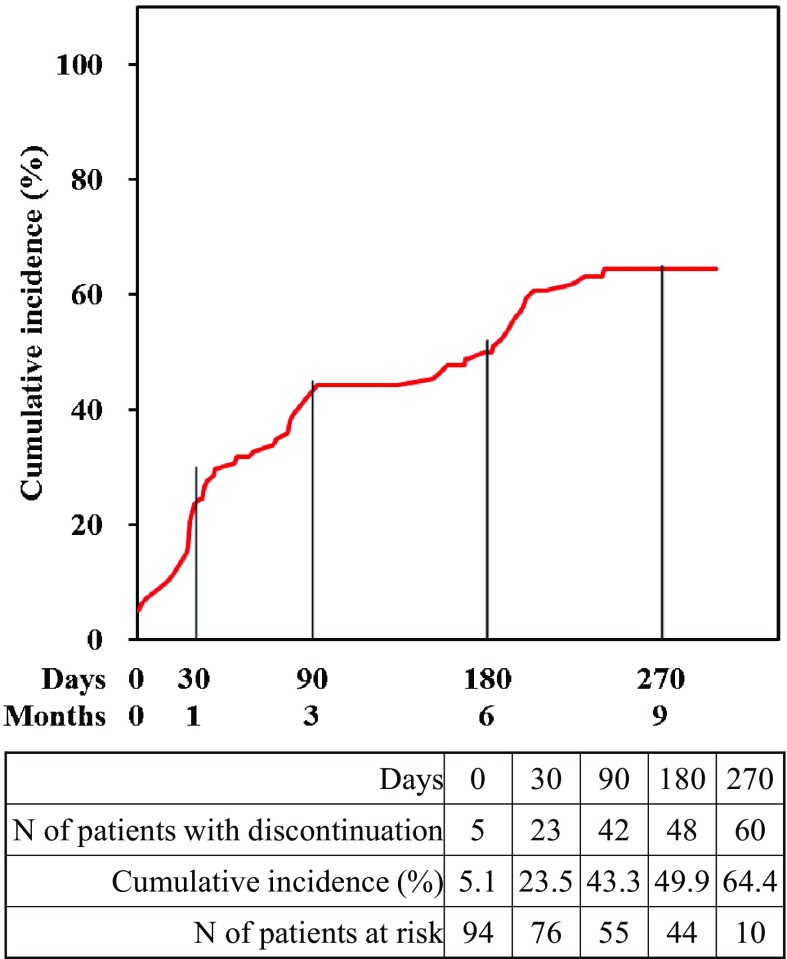
Cumulative incidence of DAPT discontinuation

The cumulative incidence of DAPT discontinuation is shown in Fig. 3. Nearly half of patients had continued DAPT at 6 months.

### Clinical outcomes

TVF occurred in 11 patients (11.2 %, 95 % CI 5.7–19.2 %) through 9 months after the index stent implantation. The upper 95 % CI for TVF of the study stent was lower than that of MULTI-LINK VISION^®^ (19.6 %), demonstrating non-inferiority of the study stent to MULTI-LINK VISION^®^.

During the 9-month follow-up, 2 patients had non-cardiac death, and no patient had MI. There was no stent thrombosis. Therefore, TVF was exclusively related to clinically indicated TVR (Table 2). Only 1 of the 11 cases had clinically indicated TVR before the 6-month angiographic follow-up (101 days after procedure) due to the recurrent angina symptom. The remaining 10 patients underwent TVR based on angiographic restenosis at the 6-month angiographic follow-up without any signs and symptoms of myocardial ischemia, such as chest pain. TVR was performed in additional 3 asymptomatic patients. However, these TVR events were not regarded as clinically indicated, because the percent diameter stenosis was <70 % by the QCA evaluation. Among 14 TVR events, 12 TVR events were related to restenosis of the study stents target lesion revascularization (TLR) events, indicating 9.2 % TLR rate. There was no new TVR event in patients who did not undergo TVR based on the angiographic finding at the 6-month follow-up. TVF tended to occur frequently in patients with relatively complex lesions, such as American Heart Association/American College of Cardiology (AHA/ACC) type B2 and C (Table 3). TVF rates tended to be lower in patients receiving stents 4.0 mm in diameter as compared with those receiving stents 3.0 or 3.5 mm in diameter, while TVF rate was very high in patients receiving stents 28 mm in length (Table 4).

**Table 2:** Clinical outcome

Outcomes	Number of patients with the event (incidence %)
TVF at 9 months (primary endpoint)	11 (11.2)
TVF at 6 months (secondary endpoint)	11 (11.2)
Any TLR at 9 months	12 (12.2)
Clinically indicated TLR at 9 months	9 (9.2)
Any TVR at 9 months	14 (14.3)
Clinically-indicated TVR at 9 months	11 (11.2)
Cardiac death at 9 months	0 (0.0)
Myocardial infarction at 9 months	0 (0.0)
Stent thrombosis at 9 months	0 (0.0)

Incidence indicated the percentage of patients with the event out of 98 patients who completed 9-month follow-up

*TVF* target vessel failure, *TLR* target lesion revascularization, *TVR* target vessel revascularization

**Table 3:** Lesion complexity and clinical outcome

AHA/ACC lesion type	A	B_1_	B_2_	C
Number of patients	6	35	48	9
TVF	0 (0 %)	2 (5.7 %)	8 (16.7 %)	1 (11.1 %)

*AHA* American Heart Association, *ACC* American College of Cardiology, *TVF* target vessel failure

**Table 4:** Stent size/length and clinical outcome

	Number of patients (%)	TVF (%)
Stent size (mm)		
3.0	38 (38.8)	5 (13.2)
3.5	29 (29.6)	4 (13.8)
4.0	31 (31.6)	2 (6.5)
Stent length (mm)		
8	1 (1.0)	0 (0.0)
13	28 (28.6)	0 (0.0)
18	38 (38.8)	6 (15.8)
23	22 (22.4)	1 (4.5)
28	9 (9.2)	4 (44.4)

### Quantitative angiographic results

Of the 98 patients who were included in the clinical efficacy analysis, 97 patients constituted the baseline quantitative angiographic analysis set excluding 1 patient with poor angiographic quality. Follow-up angiogram at 6 months after the procedure was performed and analyzed in 95 patients (97 %).

The target lesions had relatively large reference vessel diameter (2.92 ± 0.58 mm), and relatively short lesion length (13.2 ± 4.7 mm). In-stent late loss from baseline to the 6-month follow-up was 0.69 ± 0.47 mm, and binary angiographic restenosis was observed in 10 patients (10.5 %) (Table 5).

**Table 5:** Quantitative angiographic results

	Pre	Post	6 months
Number of patients analyzed	97	97	95
Lesion length (mm)	13.2 ± 4.7	–	–
Reference vessel diameter (mm)	2.92 ± 0.58	3.09 ± 0.49	2.94 ± 0.56
Minimum luminal diameter (mm)	1.08 ± 0.34	2.76 ± 0.43	2.08 ± 0.58
Percent diameter stenosis (%)	62.9 ± 9.5	10.5 ± 4.8	29.1 ± 14.6
In-stent late loss (mm)	–	–	0.69 ± 0.47
In-stent late loss index	–	–	0.42 ± 0.27
Binary restenosis	–	–	10 (10.5 %)

Binary restenosis rate indicated the percentage of patients with angiographic binary restenosis (% diameter stenosis ≥50 %) out of 95 patients who underwent 6-month angiographic follow-up

## Discussion

The current pivotal clinical study evaluating the MOMO DLC-coated coronary stent suggested its non-inferiority relative to the commercially available MULTI-LINK VISION^®^ BMS with low rates of TVF and angiographic binary restenosis (11.2 and 10.5 %, respectively), and small in-stent late loss (0.69 ± 0.47 mm). TVF tended to occur frequently in patients with relatively complex lesions, such as AHA/ACC type B2 and C. TVF rates tended to be lower in patients receiving stents 4.0 mm in diameter as compared with those receiving stents 3.0 or 3.5 mm in diameter, while TVF rate was very high in patients receiving stents 28 mm in length. As shown in Table 6, the frequency of TVF in the short stents (8–13 mm) was significantly lower than in middle (18 mm) and long (23–28 mm) stents. Therefore, the use of MOMO DLC-coated coronary stent could be an option for treating short lesions even in the DES era.

**Table 6:** Results of χ^2^-tests for the frequencies of TVF in combinations of the lesion complexity, stent size, and stent length

	*p* value
Lesion complexity	
A/B1 (2/41) vs B2/C (9/57)	0.091
Stent size	
3.0–3.5 mm (9/67) vs 4.0 mm (2/31)	0.309
3.0 mm (5/38) vs 3.5–4.0 mm (6/60)	0.629
Stent length	
8–13 mm (0/29) vs 18–28 mm (11/69)	0.022
8–18 mm (6/67) vs 23–28 mm (5/31)	0.295

In-stent late loss at the 6-month follow-up visit seemed to be lower than that reported for commercially available coronary stents [12, 13]. The binary restenosis rate of MOMO DLC-coated coronary stent also seemed to be lower than that reported for MULTI-LINK VISION^®^ (15.7 %) [13]. Thus, it was suggested that the design and DLC coating of the study device effectively prevented neo-intimal hyperplasia and restenosis.

The number of patients enrolled in this study was too small to evaluate stent thrombosis. Nonetheless, no stent thrombosis was observed in the present study. Similarly, the first-in-man study of the MOMO DLC-coated stent (a non-randomized open-label study conducted in three facilities in the United Kingdom and Germany) revealed that none of the 40 patients who received this stent developed stent thrombosis during the 6-month follow-up period [14]. The notion of thromboresistance of DLC-coated stent could be supported by the in vitro and in vivo experimental results.

**Fig. 4:**
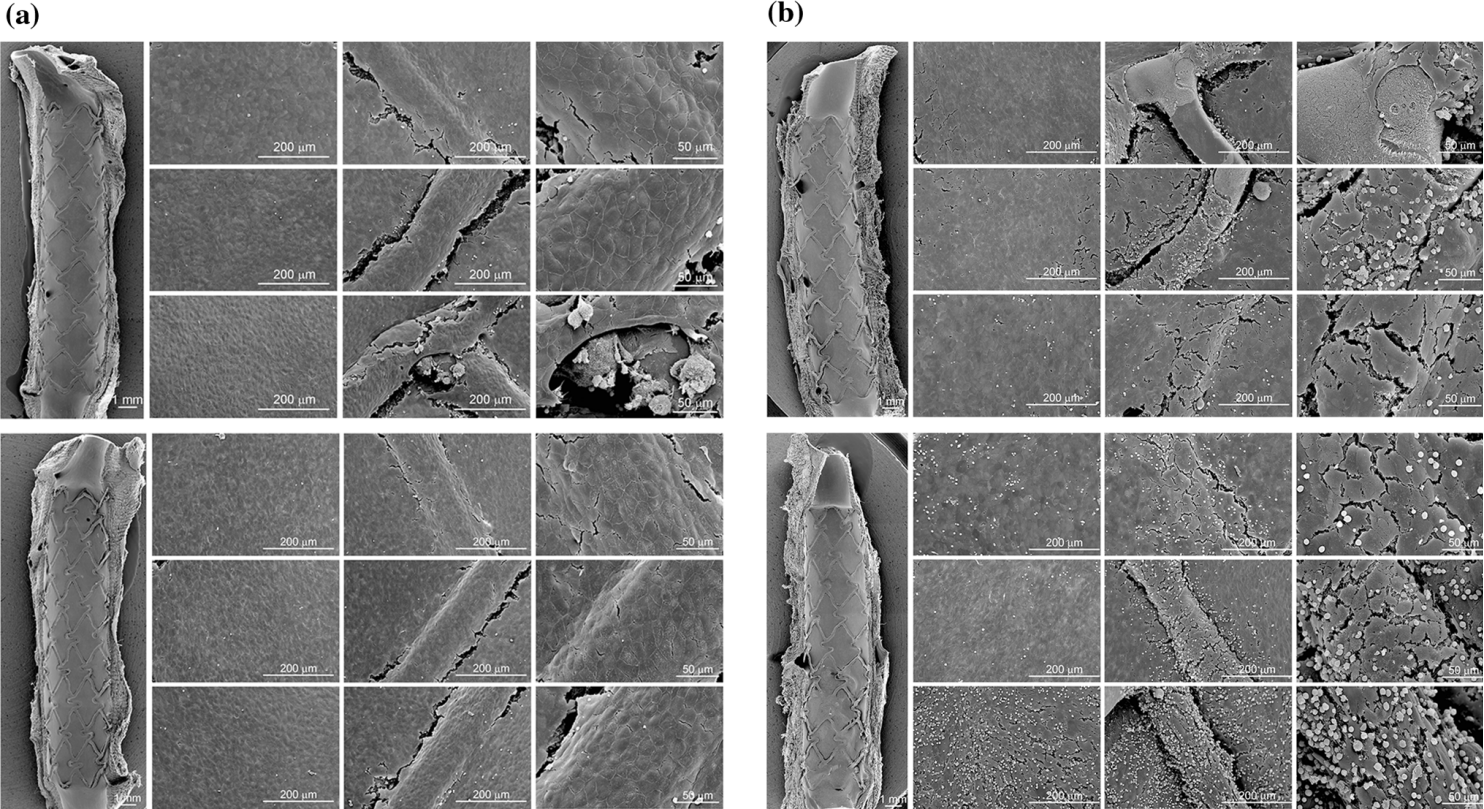
SEM images of the stent surfaces implanted 5 days to RCA: **a** MOMO DLC-coated coronary stent; **b** control stent (non-coated stent)

The in vitro tests to evaluate hemocompatibility of the DLC coating were conducted with the PTFE artificial vessel (GORE-TEX^®^ ePTFE Graft II from W. L. Gore and Associates, Inc.) as control, and results are summarized in Table 7. From the hemocompatibility tests, the number of platelet adhesion to the surface of MOMO DLC-coated coronary stent was significantly smaller than that of the PTFE artificial vessel, and the comparable effects of the DLC coating to the blood coagulation system were also seen in the thrombin–antithrombin III complex measurement. Moreover, in a pig model with a coronary artery implanted with a DLC-coated stent, scanning electron microscopy of the inner surface of the stent revealed very little platelet, and inflammatory cell adhesion as well as fibrin deposition to the DLC-coated surfaces in contrast to the significant adhesion to non-coated stent surfaces (Fig. 4; Table 8). The DLC coating of the study device consists of carbon only and is, therefore, chemically inactive. This may suppress the cell-stent surface interactions promoting thrombosis [Summary TEchnical Documentation (STED) submitted to PMDA].

**Table 7:** Results of hemocompatibility tests of the MOMO DLC-coated coronary stent

	MOMO DLC-coated coronary stent	Control (GORE-TEX^®^ ePTFE Graft II)	*p* value
Number of platelet adhesion (cells/2500 μm^2^)	1.9 ± 2.0	10.1 ± 3.3	<0.01
Thrombin–antithrombin III complex measurement (μg/L)	6.6 ± 1.9	7.9 ± 2.6	Not significant

**Table 8:** Stent struts with fibrin and red blood cell from histological analysis of in vivo test of the MOMO DLC-coated coronary stent

	Duration (days)	MOMO DLC-coated coronary stent	Control stent (non-coated stent)	*p* value
Stent struts with fibrin deposition (%)	3	51.70 ± 24.53	87.61 ± 14.94	<0.05
	5	66.37 ± 24.03	83.10 ± 15.85	<0.05
Stent struts with red blood cell deposition (%)	3	17.58 ± 15.67	23.43 ± 10.82	Not significant
	5	33.89 ± 25.30	34.94 ± 20.62	Not significant

The thromboresistance of DLC-coated stent might be beneficial in the perioperative periods of surgical procedures after coronary stent implantation, when antiplatelet therapy is often temporarily discontinued. Furthermore, the DLC coating reduces elution of metal ions from the stent surface and corrosion of the metal of the stent as confirmed by in vitro tests, provoking less inflammatory reactions in the vessel wall, and potentially ameliorating the neo-atherosclerosis formation several years after BMS implantation [15, 16]. Moreover, since the DLC coating of the MOMO DLC-coated coronary stent is highly durable against dilation and pulse beating. At least 10 years pulsatile durability of the DLC coating of the MOMO was confirmed by accelerated durability test in accordance with ASTM F2477-06. Thus, it could be expected that these beneficial effects of this stent will persist for years. A large-scale long-term post-marketing study of MOMO DLC-coated coronary stent including acute coronary syndrome is required to demonstrate its efficacy in preventing early stent thrombosis and late adverse events of the currently available BMS.

## Study limitations

1. This study used the pivotal study data of MULTI-LINK VISION^®^ coronary stent as the historical control. However, we did not assess the clinical and procedural differences between this study and the historical control study.
2. In this study, nearly half of patients had continued DAPT at 6 months, as shown in Fig. 3. Therefore, we could not evaluate the safety of the MOMO DLC-coated coronary stent with ultrashort duration (1 month) of DAPT or even with no APT.

## Conclusion

The current pivotal clinical study evaluating the new MOMO DLC-coated coronary stent suggested its low rates of TVF and angiographic binary restenosis, and small in-stent late loss, although the data were considered preliminary considering the small sample size and single arm study design.
